# Improved Multitarget Tracking in Clutter Using Bearings-Only Measurements

**DOI:** 10.3390/s18061772

**Published:** 2018-06-01

**Authors:** Yifang Shi, Mengfan Xue, Yuemin Ding, Dongliang Peng

**Affiliations:** 1 School of Automation, Hangzhou Dianzi University, Xiasha Higher Education Zone, 2nd Street, Hangzhou 310018, China; syf2008@hdu.edu.cn (Y.S.); xuemf@hdu.edu.cn (M.X.); dlpeng@hdu.edu.cn (D.P.); 2 School of Computer Science and Engineering, Tianjin University of Technology, 391 Bingshuixi Road, Xiqing District, Tianjin 300384, China

**Keywords:** bearings-only, multitarget tracking, measurement-origin-uncertainty, measurement nonlinearity, Gaussian mixture measurements-cardinality probability hypothesis density

## Abstract

Multitarget tracking in clutter using bearings-only measurements is a challenging problem. In this paper, a performance improved nonlinear filter is proposed on the basis of the Random Finite Set (RFS) theory and is named as Gaussian mixture measurements-based cardinality probability hypothesis density (GMMbCPHD) filter. The GMMbCPHD filter enables to address two main issues: measurement-origin-uncertainty and measurement nonlinearity, which constitutes the key problems in bearings-only multitarget tracking in clutter. For the measurement-origin-uncertainty issue, the proposed filter estimates the intensity of RFS of multiple targets as well as propagates the posterior cardinality distribution. For the measurement-origin-nonlinearity issue, the GMMbCPHD approximates the measurement likelihood function using a Gaussian mixture rather than a single Gaussian distribution as used in extended Kalman filter (EKF). The superiority of the proposed GMMbCPHD are validated by comparing with several state-of-the-art algorithms via intensive simulation studies.

## 1. Introduction

Multitarget tracking in clutter [1] is an interesting but difficult problem needed to be investigated, especially when only bearings-only measurements are available. Two main issues should be addressed: measurement-origin-uncertainty [2] and measurement nonlinearity [3]. The measurement-origin-uncertainty shows the common situation that one cannot tell a measurement originated from a target or clutter. As the coordinates of the tracker are usually different from those of the measurements, different levels of measurement nonlinearity arises, such as, the bearings-only measurement shows high-level nonlinearity while the range-bearing measurement gives relative low-level nonlinearity. Besides, targets may not be detected by the passive sensors thus miss-detection problem needs to be considered. Furthermore, the target tracking using bearings-only measurements is further complicated by the observability problem, in which the motion of the observer is suggested to outmaneuver the targets in order to satisfy the observability condition [4,5].

For multitarget tracking in clutter, the measurement-origin-uncertainty problem has been addressed by many classical and emerging methods. One popular traditional approach is the joint probability data association (JPDA) [1,6]. It enumerates and probabilistically evaluates every feasible measurement-to-target association event, and the target states are then estimated by using the marginal association probability. However, one main limitation of JPDA is that it can only track known and fixed number of targets. To override this limitation, joint integrated probability data association (JIPDA) was proposed in [7], which recursively calculates the probability of target existence as a track quality measure to enable track management, as a result, the number of tracked targets can be effectively estimated. The abovementioned methods all utilize the feasible joint events to effectively address the measurement-origin-uncertainty problem in multitarget tracking; however, it suffers from heavy computational load and the time complexity increases exponentially along with the number of tracks and measurements involved in the cluster. To alleviate the computational load, the linear multitarget integrated probability data association (LMIPDA) [3] is then proposed to modulate the clutter measurement density by considering the possible contributions of targets being followed by other tracks, in which the numerical complexity is linear in the number of targets and the number of measurements. Multiple hypothesis tracker (MHT) [2,8,9] is another classical tracker for multitarget tracking in clutter. Though many versions of MHT have been developed, most of them can be divided into two categories: track-oriented and measurement-oriented. In terms of the tracking accuracy, the MHT-based methods usually outperform than the JPDA-based methodologies; however, the computational complexity of MHT is much heavier than JPDA. To balance the computational load and tracking accuracy, the emerging Random Finite Set (RFS)-based methods provide a prominent alternative recently [10,11]. The key idea of RFS-based methods is to model the multitarget states and corresponding measurements as a state set and a measurement set, respectively, in which the measurement set is utilized to estimate the multitarget state set and the measurement-to-target association is thus tactfully avoided.

The measurement nonlinearity [12] issue occurs in many practical situations when the coordinates of target states are different from those of measurements, as a consequence, different types of measurements have different levels of nonlinearity. Typically, the nonlinearity of bearings-only measurements usually gives higher level of nonlinearity than that of range-bearing measurements. To resolve the measurement nonlinearity problem, many techniques have been proposed. The most popular one is the extended Kalman filter (EKF) [12], which linearizes the measurement nonlinear function at the predicted target state using Taylor expansion, and the subsequent update procedure is similar with that of Kalman filter (KF) [12]. Another well-known nonlinear filter is the particle filter (PF) [13] which has been developed into many more advanced versions since proposed, such as sampling importance resampling (SIR), regularized particle filter (RPF) [4] and so on. The main difference between EKF and PF is that the posterior density of target state is modeled by a Gaussian distribution in the EKF while modeled using amounts of weighted particles in the PF. Thus the computational resources that the PF consumes are quite larger than that of EKF. Besides, there are also many other types of filters to deal with the nonlinearity of measurements, just name a few, unscented Kalman filter (UKF) [14], cubature Kalman filter (CKF) [15] and ensemble Kalman filter (EnKF) [16] etc.

Recently, in order to address multitarget tracking in clutter using bearings-only measurements, an improved RFS-based filter, called the Gaussian mixture measurements-based probability hypothesis density (GMMbPHD), was proposed in [17]. The probability hypothesis density (PHD) [18] in GMMbPHD is the first moment approximation of the density of target states set. As there is no close form solution of the PHD filter, approximated solutions such as sequential monte carlo (SMC) [19,20] and Gaussian mixture (GM) are usually two suggested implementation methods. Though SMC method delivers relative higher tracking accuracy than the GM method, unfortunately, it is difficult to extract estimated target states and usually computationally-dense demanding. Thus GMMbPHD improves the existing GM-based PHD (GMbPHD) [18] by using the Gaussian mixture measurements (GMM) technique [21], which models the likelihood function of nonlinear measurements using Gaussian mixtures instead of a single Gaussian probability density function used in the EKF. Compared with GMbPHD with EKF (GMbPHDwEKF) and GMbPHD with UKF (GMbPHDwUKF), GMMbPHD shows significant improvement on position accuracy. However, the GMMbPHD algorithm focuses on the position improvement and neglects the cardinality. As a result, in order to fully improve the tracking performance on both position and cardinality accuracy, an further improved filter, termed as the Gaussian mixture measurements-based cardinality probability hypothesis density (GMMbCPHD), is proposed in this paper. Inspired from the idea of the Gaussian mixture measurement methodology, the GMMbCPHD algorithm approximates the likelihood function of the bearings-only measurements by a Gaussian mixture of numbers of refined Gaussian probability density functions, thereafter, rederives the update procedure of the cardinality and intensity density of the multitarget state set, eventually, the improved formulations on both the localization and position intensity update are obtained. For the sake of verifying the effectiveness and superiority of the proposed filter, numbers of classical experiments are simulated to compare the GMMbCPHD with several state-of-the-art algorithms, i.e., GMbPHDwEKF, GMbPHDwUKF, GMMbPHD, GM-based cardinality PHD with EKF (GMbCPHDwEKF) and GM-based cardinality PHD with UKF (GMbCPHDwUKF), the tracking performance is shown in terms of the optimal subpattern assignment (OSPA) [22] as well as OSPA localization and OSPA cardinality.

In Section 2, the target motion model, measurement model and clutter model are presented. The proposed GMMbCPHD filter is given in detail in Section 3. Simulation studies are presented in Section 4. Finally, a conclusion is proposed in Section 5.

## 2. Bearings-Only Multitarget Tracking Models

### 2.1. Target Motion Model

In this paper, we assume that the motion of each target follows the continuous white noise acceleration motion model [1,12] given by

(1) $$x_{k}^{t}=\Phi x_{k-1}^{t}+\nu_{k-1},$$

where the state propagation matrix Φ is time-invariant,

(2) $$\Phi =\left[\begin{array}{cc} 1 & T\\ 0 & 1 \end{array}\right]⊗I_{2},$$

and $\nu_{k-1}$ is a sequence of zero mean, white Gaussian process noises with covariance

(3) $$Q_{k-1}=q\left[\begin{array}{cc} T^{4}/4 & T^{3}/2\\ T^{3}/2 & T^{2} \end{array}\right]⊗I_{2}.$$

Besides, *T* is the sampling time, $I_{2}$ is the 2×2 identity matrix, and *q* is the power spectral density (PSD) [1]. Please note that the target *t* with state $x_{k}^{t}$ is presented as

(4) $$x_{k}^{t}={\left[x_{k}^{t},y_{k}^{t},{\dot{x}}_{k}^{t},{\dot{y}}_{k}^{t}\right]}^{\prime },$$

where $\left(x_{k}^{t},y_{k}^{t}\right)$ and velocity $\left({\dot{x}}_{k}^{t},{\dot{y}}_{k}^{t}\right)$ are position and velocity at time *k*, respectively.

In a target state set $X_{k}=\left\{x_{k,1}^{t},\ldots ,x_{k,N_{k}}^{t}\right\}$, the number of targets $N_{k}$ can be random value and the sequence of target states in the set can also be random. In this paper, we assume that χ denote the single target state space and F(χ) denote the set of all finite subsets of χ, i.e., $x_{k,1}^{t},\ldots ,x_{k,N_{k}}^{t}\in \chi$ with $X_{k}\in F\left(\chi \right)$.

### 2.2. Bearings-Only Measurement Model

Let $x_{k}^{s}$ denote the sensor state shown as $x_{k}^{s}={\left[x_{k}^{s},y_{k}^{s},{\dot{x}}_{k}^{s},{\dot{y}}_{k}^{s}\right]}^{\prime }$. At each sampling time *k*, we assume that each target *t* can be detected with the probability $P_{D,k}$. Using $\theta_{k}^{t}$ to denote the bearings-only measurement of target *t* if it is detected. The measurement generated by the passive sensor is represented as

(5) $$z_{k}≜\theta_{k}^{t}=h\left(x_{k}^{t},x_{k}^{s}\right)+\varpi_{k},$$

where

(6) $$h\left(x_{k}^{t},x_{k}^{s}\right)=\tan^{-1}\left(\frac{x_{k}^{t}-x_{k}^{s}}{y_{k}^{t}-y_{k}^{s}}\right),$$

and $\varpi_{k}$ is white Gaussian measurement noise with zero mean and covariance

(7) $$R_{k}=\sigma_{\theta }^{2},$$

which is uncorrelated with $\nu_{k}$. Obviously, the measurement set $\Theta_{k}\left(x_{k}^{t}\right)$ generated by each target *t* can be either $\left\{\theta_{k}^{t}\right\}$ or ∅ where denote that the target *t* is detectable or not detected, respectively.

### 2.3. Clutter Measurement Model

In practical situations, some false measurements originated from clutter may also be detected by the sensor and form an RFS $K_{k}$ at each time *k*. These false measurements are always assumed to be uniformly distribute in the sensor detection space. Finally, the bearings-only measurements RFS $Z_{k}$ at time *k* can be expressed as

(8) $$Z_{k}=K_{k}\cup \left[\underset{x\in X_{k}}{⋃}\Theta_{k}\left(x\right)\right].$$

Furthermore, we use $Z^{k}=\left\{Z_{1},\ldots ,Z_{k}\right\}$ to denote the measurement collection from time 1 to time *k*.

## 3. The Proposed GMMbCPHD Filter

In the framework of the RFS-based methods, the Bayesian recursion of multitarget posterior density propagates in time as

(9) $$p_{k|k-1}\;\left(X_{k}|Z^{k-1}\;\right)=\int \;f_{k|k-1}\;\left(X_{k}|X\;\right)p_{k-1}\;\left(X|Z^{k-1}\;\right)\;\mu_{s}\;\left(dX\;\right),$$

(10) $$p_{k}\left(X_{k}|Z^{k}\right)=\frac{g_{k}\left(Z_{k}|X_{k}\right)p_{k|k-1}\left(X_{k}|Z^{k-1}\right)}{\int g_{k}\left(Z_{k}|X\right)p_{k|k-1}\left(X|Z^{k-1}\right)\mu_{s}\left(dX\right)},$$

where $f_{k|k-1}\left(X_{k}|X\right)$ denotes the transition density of multitarget state and $g_{k}\left(Z_{k}|X_{k}\right)$ represents the measurement likelihood. Furthermore, $p_{k}\left(X_{k}|Z^{k}\right)$ denotes the multitarget state posterior density, with $\mu_{s}\;\left(dX\right)$ denoting an proper reference measure on F(χ) [18].

Obviously, the recursion in (9) and (10) is difficult to be calculated. Thus a first moment approximation was proposed in [18] and named as PHD filter. The PHD propagates the intensity density of the multitarget state density and the general form is given by (without target spawning):

(11) $$\begin{array}{c} v_{k|k-1}\;\left(x\right)=\int \;P_{S,k}\;\left(\eta \right)f_{k|k-1}\;\left(x|\eta \;\right)v_{k-1}\;\left(\eta \right)d\eta +\gamma_{k}\;\left(x\right), \end{array}$$

(12) $$\begin{array}{c} v_{k|k}\left(x\right)=\left[1-P_{D,k}\left(x\right)\right]v_{k|k-1}\left(x\right)+\sum_{z\in Z_{k}}\frac{P_{D,k}\left(x\right)g_{k}\left(z|x\right)v_{k|k-1}\left(x\right)}{\kappa_{k}\left(z\right)+\int P_{D,k}\left(x\right)g_{k}\left(z|x\right)v_{k|k-1}\left(x\right)dx}, \end{array}$$

where $v_{k|k-1}\left(x\right)$ and $v_{k|k}\left(x\right)$ denote the predicted intensity and the updated intensity, respectively, $P_{S,k}\left(\eta \right)$ is the target survival probability from time k−1 to *k*. $f_{k|k-1}\left(x|\eta \right)$ and $g_{k}\left(z|x\right)$ represent the single target transition density and measurement likelihood function, respectively. $\gamma_{k}\left(x\right)$ denotes the prior intensity of spontaneous target births and $\kappa_{k}\left(z\right)$ is the clutter intensity at time *k*.

To improve the estimation accuracy of the number of targets, except the intensity density, the CPHD [23] also propagates the cardinality density:

(13) $$p_{k|k-1}\left(n\right)=\sum_{i=0}^{n}p_{\gamma ,k}\left(n-i\right)\sum_{j=i}^{\infty }C_{i}^{j}\frac{{\langle P_{S,k},v_{k|k-1}\rangle }^{i}{\langle 1-P_{S,k},v_{k|k-1}\rangle }^{j-i}}{{\langle 1,v_{k|k-1}\rangle }^{j}}p_{k-1|k-1}\left(j\right),$$

(14) $$v_{k|k-1}\left(x\right)=\int P_{S,k}\left(x\right)f_{k|k-1}\left(x_{k}|x_{k-1}\right)v_{k-1}\left(x\right)dx_{k-1}+\gamma_{k}\left(x\right),$$

(15) $$p_{k|k}\left(n\right)=\frac{Y_{k}^{0}\left[v_{k|k-1},Z_{k}\right]\left(n\right)}{\langle Y_{k}^{0}\left[v_{k|k-1},Z_{k}\right],p_{k|k-1}\rangle }p_{k|k-1}\left(n\right),$$

(16) $$\begin{array}{c} v_{k|k}\left(x\right)=\frac{\langle Y_{k}^{1}\left[v_{k|k-1},Z_{k}\right],p_{k|k-1}\rangle }{\langle Y_{k}^{0}\left[v_{k|k-1},Z_{k}\right],p_{k|k-1}\rangle }\left[1-P_{D,k}\left(x_{k}\right)\right]v_{k|k-1}\left(x\right)\;\\ \;+\sum_{z_{k}\in Z_{k}}\frac{\langle Y_{k}^{1}\left[v_{k|k-1},Z_{k}\backslash \left\{z_{k}\right\}\right],p_{k|k-1}\rangle }{\langle Y_{k}^{0}\left[v_{k|k-1},Z_{k}\right],p_{k|k-1}\rangle }\psi_{k}\left(x\right)v_{k|k-1}\left(x\right), \end{array}$$

(17) $$Y_{k}^{u}\left[v,Z\right]\left(n\right)=\sum_{i=0}^{\min\left(\left|Z\right|,n\right)}\left(\left|Z\right|-i\right)!p_{\kappa ,k}\left(\left|Z\right|-i\right)P_{i+u}^{n}\frac{{\langle 1-P_{D,k},v_{k|k-1}\rangle }^{n-\left(i+u\right)}}{{\langle 1,v_{k|k-1}\rangle }^{n}}E_{i}\left(\Lambda_{k}\left(v,Z\right)\right),$$

(18) $$\Lambda_{k}\left(v,Z\right)=\left\{\langle v_{k|k-1},\psi_{k}\rangle :z\in Z\right\},$$

(19) $$\psi_{k}\left(x\right)=\frac{\langle 1,\kappa_{k}\rangle }{\kappa_{k}\left(z_{k}\right)}g_{k}\left(z_{k}|x_{k}\right)P_{D,k}\left(x\right),$$

where:

$C_{i}^{j}=\frac{j!}{i!\left(j-i\right)!}$ is the binomial coefficient;
$P_{i}^{n}=\frac{n!}{\left(n-i\right)!}$ is the permutation coefficient;
$\langle \alpha ,\beta \rangle =\int \alpha \left(x\right)\beta \left(x\right)dx$ denotes the inner product defined between two real-valued functions α and β;
$E_{i}\left(Z\right)=\sum_{S\subseteq Z,\left|S\right|=i}\left(\prod_{\eta \in S}\eta \right)$ is the elementary symmetric function defined for a finite set Z of real numbers with $E_{0}\left(Z\right)=1$ by convention;
$p_{k-1|k-1}$ denotes the posterior cardinality distribution at time k−1;
$p_{\gamma ,k}$ is the prior cardinality distribution of spontaneous births at time *k*;
$p_{\kappa ,k}$ is the cardinality distribution of clutter at time *k*;
$P_{S,k}\left(x\right)$ is the probability of survival at time *k*;
$f_{k|k-1}\left(x_{k}|x_{k-1}\right)$ is the target transition density from time k−1 to time *k*;
$\gamma_{k}\left(x\right)$ is the prior intensity of spontaneous target births at time *k*;
$P_{D,k}\left(x\right)$ is the probability of detection at time *k*;
$g_{k}\left(z_{k}|x_{k}\right)$ is the likelihood function;
$\kappa_{k}\left(z\right)$ is the clutter intensity at time *k*.

### 3.1. Cardinality and Intensity Prediction of GMMbCPHD

The cardinality distribution of multitarget is propagated from time k−1 to time *k*, given by

(20) $$p_{k|k-1}\left(n\right)=\sum_{i=0}^{n}p_{\gamma ,k}\left(n-i\right)\sum_{j=i}^{\infty }C_{i}^{j}P_{S,k}^{i}{\left(1-P_{S,k}\right)}^{j-i}p_{k-1|k-1}\left(j\right).$$

Based on the Gaussian mixture assumption, the posterior intensity at time k−1 can be represented as

(21) $$v_{k-1}\left(x\right)=\sum_{i=1}^{J_{k-1}}\omega_{k-1}^{\left(i\right)}N\left(x_{k-1};{\hat{x}}_{k-1}^{\left(i\right)},J_{k-1}^{\left(i\right)}\right).$$

Assume that θ,r,c,ands denote the bearing, range, course and speed in polar coordinates, respectively. The function $\Psi \left(x;\gamma_{k}\left(\theta ,r,c,s\right)\right)$ is utilized here to interpret the transformation of the density $\gamma_{k}\left(\theta ,r,c,s\right)$ from polar coordinates to Cartesian coordinates. Then, the predicted intensity at time *k* is given by

(22) $$\begin{array}{c} v_{k|k-1}\left(x\right)=v_{S,k|k-1}\left(x\right)+\Psi \left(x;\gamma_{k}\left(\theta ,r,c,s\right)\right), \end{array}$$

where

(23) $$\begin{array}{c} v_{S,k|k-1}\left(x\right)=P_{S,k}\sum_{i=1}^{J_{k-1}}\omega_{k-1}^{\left(i\right)}N\left(x;\;{\hat{x}}_{k|k-1}^{\left(i\right)},J_{k|k-1}^{\left(i\right)}\right), \end{array}$$

(24) $$\begin{array}{c} \gamma_{k}\left(\theta ,r,c,s\right)=\omega_{k}^{b}U\;\left(\theta ;H_{\theta }\right)N\left(r;\bar{r},\sigma_{r}^{2}\right)N\left(c;\theta -\pi ,\sigma_{c}^{2}\right)N\left(s;\bar{s},\sigma_{s}^{2}\right). \end{array}$$

In Equation (23), $P_{S,k}\left(x\right)$ denotes the survived probability and is usually independent of the target state, for simplicity, it is abbreviated as $P_{S,k}$. In Equation (24), $\omega_{k}^{b}$ denotes the expected number of targets that are born at time *k*, $U\left(\theta ;H_{\theta }\right)$ denotes a uniform distribution with respect to θ over the region $H_{\theta }$ [24], $\bar{r}$ and $\sigma_{r}^{2}$ are the prior known mean range and it corresponded variance, respectively. Besides, $\bar{s}$ and $\sigma_{s}^{2}$ denotes the prior known mean speed and it corresponded variance, respectively, with $\sigma_{c}^{2}$ denoting the prior known course variance. ${\hat{x}}_{k|k-1}^{\left(i\right)}$ and $J_{k|k-1}^{\left(i\right)}$ are the predicted target state estimates and its corresponded covariance, which are calculated based on the prediction step of the Kalman filter:

(25) $$\begin{array}{c} {\hat{x}}_{k|k-1}^{\left(i\right)}=\Phi {\hat{x}}_{k-1}^{\left(i\right)}, \end{array}$$

(26) $$\begin{array}{c} J_{k|k-1}^{\left(i\right)}=\Phi J_{k|k-1}^{\left(i\right)}\Phi^{{}^{\prime }}+Q_{k-1}. \end{array}$$

A binary variable β is utilized to augment the target state so as to distinguish the surviving components from the birth components.

(27) $$\begin{array}{c} v_{k|k-1}\left(x,\beta \right)=\left\{\begin{array}{c} \sum_{i=1}^{J_{k-1}}\omega_{k|k-1}^{\left(i\right)}N\left(x;{\hat{x}}_{k|k-1}^{\left(i\right)},J_{k|k-1}^{\left(i\right)}\right),\;\beta =0\\ \Psi \left(x;\gamma_{k}\left(\theta ,r,c,s\right)\right),\;\beta =1, \end{array}\right. \end{array}$$

where $\omega_{k|k-1}^{\left(i\right)}=P_{S,k}\omega_{k-1}^{\left(i\right)}$. As mentioned in [25], in order to avoid the cardinality estimation bias, the surviving component and birth components are suggested to be separately considered.

### 3.2. GMM Model in GMMbCPHD

In the proposed GMMbCPHD filter, the measurement likelihood function $g_{k}\left(z|x\right)$ is modeled by Gaussian mixtures but not a single Gaussian distribution. As the measurement uncertainty of bearings-only measurement is non-Gaussian in Cartesian coordinates, thus the key idea of the GMM method is to model this non-Gaussian measurement uncertainty area by several refined components, where each component can be approximated by a Gaussian distribution.

We use the range interval $\left[r_{k,\min},r_{k,\max}\right]$ and received measurement $\theta_{k}^{t}$ along with the standard deviation $\sigma_{\theta }$ to illustrate the measurement uncertainty in Cartesian coordinates. Then the range interval is divided into $A_{k}$ subintervals, given by [21,26]

(28) $$\frac{r_{k,a+1}}{r_{k,a}}=\tau_{k};\;a=1,\ldots ,A_{k},$$

where

(29) $$\tau_{k}={\left(\frac{r_{k,\max}}{r_{k,\min}}\right)}^{1/A_{k}}.$$

Please note that each component *a* is determined by $r_{k,a},r_{k,a+1},\theta_{k}^{t}-\sigma_{\theta },\theta_{k}^{t}+\sigma_{\theta }$ in polar coordinates. Based on the information of $\bar{r}=\left(r_{k,a}+r_{k,a+1}\right)/2$ and $\Delta r=\left(r_{k,a+1}-r_{k,a}\right)/2$, the probability density function of measurement component *a* is approximated by a Gaussian distribution with mean ${\hat{z}}_{k,a}$ and covariance $R_{k,a}$ defined in Cartesian coordinates, obtained by

(30) $${\hat{z}}_{k,a}=\left[\begin{array}{c} x_{k}^{s}\\ y_{k}^{s} \end{array}\right]+\bar{r}\left[\begin{array}{c} \sin\left(\theta_{k}^{t}\right)\\ \cos\left(\theta_{k}^{t}\right) \end{array}\right],$$

(31) $$R_{k,a}=\phi \left[\begin{array}{cc} {\left(\Delta r\right)}^{2} & 0\\ 0 & {\bar{r}}^{2}\sigma_{\theta }^{2} \end{array}\right]\phi^{\prime },$$

where the transformation matrix

(32) $$\phi =\left[\begin{array}{cc} \sin\left(\theta_{k}^{t}\right) & -\cos\left(\theta_{k}^{t}\right)\\ \cos\left(\theta_{k}^{t}\right) & \sin\left(\theta_{k}^{t}\right) \end{array}\right].$$

As the area of each component is different, thus the component weight is chosen to be proportional to the area, given by [26]

(33) $$\lambda_{k,a}=\frac{\sqrt{\det\left(R_{k,a}\right)}}{\sum_{a=1}^{A_{k}}\sqrt{\det\left(R_{k,a}\right)}},$$

and

(34) $$\sum_{a=1}^{A_{k}}\lambda_{k,a}=1.$$

Finally, the likelihood function $\left(\beta =0\right)$ is approximated using a Gaussian mixture

(35) $$\begin{array}{c} g_{k}\left(z|x,\beta =0\right)\approx C_{k}\sum_{a=1}^{A_{k}}\lambda_{k,a}N\;\left({\hat{z}}_{k,a};Hx_{k},R_{k,a}\right), \end{array}$$

where the observation matrix $H=\left[\begin{array}{cccc} 1 & 0 & 0 & 0\\ 0 & 1 & 0 & 0 \end{array}\right]$, with the constant $C_{k}$ calculated by [27]

(36) $$C_{k}=\int_{r_{k,\min}}^{r_{k,\max}}rdr=\frac{r_{k,\max}^{2}-r_{k,\min}^{2}}{2}.$$

From the measurement model defined in Equation (3), we can know the measurement noise follows a Gaussian distribution, therefore the likelihood function defined in the polar coordinates $\left(\beta =1\right)$ can be obtained by

(37) $$\begin{array}{c} g_{k}\left(z|x,\beta =1\right)=N\;\left(\theta_{k}^{t};\theta ,\sigma_{\theta }^{2}\right). \end{array}$$

### 3.3. Cardinality and Intensity Update of GMMbCPHD

The new targets are always assumed to be detected when they are birth. For the sake of simplicity, the detection probability for surviving target is assumed to be independent from their state, which results in the definition as follows

(38) $$P_{D,k}\left(x,\beta \right)=\left\{\begin{array}{c} P_{D,k},\;\beta =0\\ 1,\;\beta =1. \end{array}\right.$$

To make the mathematical derivation procedure friendlier, the notations defined below are necessary

(39) $$\chi =\frac{\langle Y_{k}^{1}\left[v_{k|k-1},Z^{k}\right],p_{k|k-1}\rangle }{\langle Y_{k}^{0}\left[v_{k|k-1},Z^{k}\right],p_{k|k-1}\rangle },$$

(40) $$\chi \left(z\right)=\frac{\langle Y_{k}^{1}\left[v_{k|k-1},Z^{k}\backslash \left\{z_{k}\right\}\right],p_{k|k-1}\rangle }{\langle Y_{k}^{0}\left[v_{k|k-1},Z^{k}\right],p_{k|k-1}\rangle },$$

(41) $$p_{k|k}\left(n\right)=\frac{Y_{k}^{0}\left[v_{k|k-1},Z^{k}\right]\left(n\right)}{\langle Y_{k}^{0}\left[v_{k|k-1},Z^{k}\right],p_{k|k-1}\rangle }p_{k|k-1}\left(n\right),$$

(42) $$v_{k|k}\left(x,0\right)=\left[1-P_{D,k}\right]\chi v_{k|k-1}\left(x,\beta =0\right)+\sum_{z_{k}\in Z^{k}}\chi \left(z\right)\psi_{k}\left(x,\beta =0\right)v_{k|k-1}\left(x,\beta =0\right),$$

(43) $$v_{k|k}\left(x,\beta =1\right)=\sum_{z_{k}\in Z^{k}}\chi \left(z\right)\psi_{k}\left(x,\beta =1\right)v_{k|k-1}\left(x,\beta =1\right),$$

where

(44) $$Y_{k}^{u}\left[v,Z\right]\left(n\right)=\sum_{i=0}^{\min\left(\left|Z\right|,n\right)}\left(\left|Z\right|-i\right)!p_{\kappa ,k}\left(\left|Z\right|-i\right)P_{i+u}^{n}\frac{{\langle 1-P_{D,k},v_{k|k-1}\rangle }^{n-\left(i+u\right)}}{{\langle 1,v_{k|k-1}\rangle }^{n}}E_{i}\left(\Lambda_{k}\left(v,Z\right)\right),$$

(45) $$\Lambda_{k}\left(v,Z\right)=\left\{\langle v_{k|k-1},\psi_{k}\rangle :z\in Z\right\},$$

(46) $$\psi_{k}\left(x,\beta \right)=\frac{\langle 1,\kappa_{k}\rangle }{\kappa_{k}\left(z_{k}\right)}g_{k}\left(z_{k}|x,\beta \right)P_{D,k}\left(x,\beta \right).$$

The inner product with respect to $v_{k|k-1}$ and $\psi_{k}$ is defined and obtained by

(47) $$\begin{array}{c} \langle v_{k|k-1},\psi_{k}\rangle =\;\int \int \;v_{k|k-1}\left(x,\beta \right)\psi_{k}\left(x,\beta \right)dxd\beta \;\\ \;=\int \sum_{\beta =0}^{1}v_{k|k-1}\left(x,\beta \right)\psi_{k}\left(x,\beta \right)dx\;\\ \;=\int \sum_{i=1}^{J_{k-1}}\omega_{k|k-1}^{\left(i\right)}N\left(x;{\hat{x}}_{k|k-1}^{\left(i\right)},J_{k|k-1}^{\left(i\right)}\right)\frac{\langle 1,\kappa_{k}\rangle }{\kappa_{k}\left(z_{k}\right)}g_{k}\left(z_{k}|x,\beta =0\right)P_{D,k}\left(x,\beta =0\right)\;\\ \;+\Psi \left(x;\gamma_{k}\left(\theta ,r,c,s\right)\right)\frac{\langle 1,\kappa_{k}\rangle }{\kappa_{k}\left(z_{k}\right)}g_{k}\left(z_{k}|x\right)P_{D,k}\left(x,\beta =1\right)dx\;\\ \;\approx \sum_{i=1}^{J_{k-1}}\omega_{k|k-1}^{\left(i\right)}\frac{\langle 1,\kappa_{k}\rangle }{\kappa_{k}\left(z_{k}\right)}C_{k}\sum_{a=1}^{A_{k}}\lambda_{k,a}P_{D,k}\int N\;\left({\hat{z}}_{k,a};Hx_{k},R_{k,a}\right)N\left(x;{\hat{x}}_{k|k-1}^{\left(i\right)},J_{k|k-1}^{\left(i\right)}\right)dx\;\\ \;+\frac{\langle 1,\kappa_{k}\rangle }{\kappa_{k}\left(z_{k}\right)}\frac{\omega_{k}^{b}}{2\pi }\sum_{a=1}^{A_{k}}\lambda_{k,a}\int N\left(x;{\tilde{x}}_{k}\left(\theta_{k}^{t}\right),{\tilde{J}}_{k}\left(\theta_{k}^{t}\right)\right)dx\;\\ \;=\frac{\langle 1,\kappa_{k}\rangle }{\kappa_{k}\left(z_{k}\right)}\left(\frac{\omega_{k}^{b}}{2\pi }+C_{k}P_{D,k}\sum_{i=1}^{J_{k-1}}\sum_{a=1}^{A_{k}}\omega_{k|k-1}^{\left(i\right)}\lambda_{k,a}q_{k}^{\left(i\right)}\left({\hat{z}}_{k,a}\right)\right), \end{array}$$

where $q_{k}^{\left(i\right)}\left({\hat{z}}_{k,a}\right)=N\left({\hat{z}}_{k,a};H{\hat{x}}_{k|k-1}^{\left(i\right)},S_{k|k-1}^{\left(i\right)}\right)$ denotes the likelihood of measurement component ${\hat{z}}_{k,a}$ with respect to component *i*, and the innovation covariance:

(48) $$S_{k|k-1}^{\left(i\right)}=HJ_{k|k-1}^{\left(i\right)}H^{{}^{\prime }}+R_{k,a},$$

and the updated target states and corresponding covariance are given by:

(49) $${\hat{x}}_{k|k}^{\left(i,a\right)}={\hat{x}}_{k|k-1}^{\left(i\right)}+K_{k}^{\left(i\right)}\left({\hat{z}}_{k,a}-H{\hat{x}}_{k|k-1}^{\left(i\right)}\right),$$

(50) $$J_{k|k}^{\left(i,a\right)}=J_{k|k-1}^{\left(i\right)}-K_{k}^{\left(i\right)}HJ_{k|k-1}^{\left(i\right)},$$

with the Kalman gain:

(51) $$K_{k}^{\left(i\right)}=J_{k|k-1}^{\left(i\right)}H^{{}^{\prime }}{\left(S_{k|k-1}^{\left(i\right)}\right)}^{-1}.$$

In the third step of Equation (47), as both likelihood function $g_{k}\left(z|x,\beta =1\right)$ and the target birth model $\gamma_{k}\left(\theta ,r,c,s\right)$ are given in the same polar coordinates, we have:

(52) $$g_{k}\left(z|x\right)\Psi \left(x;\gamma_{k}\left(\theta ,r,c,s\right)\right)=\Psi \left(x;g_{k}\left(z|x\right)\gamma_{k}\left(\theta ,r,c,s\right)\right).$$

Then,

(53) $$\begin{array}{c} \varphi_{k}\left(\theta ,r,c,s\right)≜g_{k}\left(z|x\right)\gamma_{k}\left(\theta ,r,c,s\right)\;\\ =N\;\left(\theta_{k}^{t};\theta ,\sigma_{\theta }^{2}\right)\omega_{k}^{b}U\;\left(\theta ;H_{\theta }\right)N\left(r;\bar{r},\sigma_{r}^{2}\right)N\left(c;\theta -\pi ,\sigma_{c}^{2}\right)N\left(s;\bar{s},\sigma_{s}^{2}\right)\;\\ =\omega_{k}^{b}\frac{1_{H}\left(\theta \right)}{V_{H}}N\;\left(\theta ;\theta_{k}^{t},\sigma_{\theta }^{2}\right)N\left(r;\bar{r},\sigma_{r}^{2}\right)N\left(c;\theta -\pi ,\sigma_{c}^{2}\right)N\left(s;\bar{s},\sigma_{s}^{2}\right)\;\\ \approx \frac{\omega_{k}^{b}}{2\pi }N\;\left(\theta ;\theta_{k}^{t},\sigma_{\theta }^{2}\right)N\left(r;\bar{r},\sigma_{r}^{2}\right)N\left(c;\theta -\pi ,\sigma_{c}^{2}\right)N\left(s;\bar{s},\sigma_{s}^{2}\right), \end{array}$$

where $1_{H}\left(\theta \right)$ denotes the indicator function of the bearings-only measurement space region $H_{\theta }$ and $V_{H}$ is the volume of $H_{\theta }$. Besides, the following approximation is made:

(54) $$1_{H}\left(\theta \right)N\;\left(\theta ;\theta_{k}^{t},\sigma_{\theta }^{2}\right)\approx N\;\left(\theta ;\theta_{k}^{t},\sigma_{\theta }^{2}\right).$$

The transformation function $\Psi \left(x;\varphi_{k}\left(\theta ,r,c,s\right)\right)$ is used to transform the function $\varphi_{k}\left(\theta ,r,c,s\right)$ from polar coordinate to Cartesian coordinate, which is obtained by:

(55) $$\Psi \left(x;\varphi_{k}\left(\theta ,r,c,s\right)\right)\approx \frac{\omega_{k}^{b}}{2\pi }\sum_{a=1}^{A_{k}}\lambda_{k,a}N\left(x;{\tilde{x}}_{k}\left(\theta_{k}^{t}\right),{\tilde{J}}_{k}\left(\theta_{k}^{t}\right)\right),$$

where the weight $\lambda_{k,a}$ is calculated by Equation (33), the position component of ${\tilde{x}}_{k}\left(\theta_{k}^{t}\right)$ and ${\tilde{J}}_{k}\left(\theta_{k}^{t}\right)$ are obtained by Equations (56) and (57), respectively, and the velocity component can be approximately calculated in the way in [28,29]:

(56) $${\tilde{x}}_{k}\left(\theta_{k}^{t}\right)=\left[\begin{array}{c} x_{k}^{s}+\bar{r}\sin\left(\theta_{k}^{t}\right)\;\\ y_{k}^{s}+\bar{r}\cos\left(\theta_{k}^{t}\right)\;\\ \bar{s}\sin\left(\theta_{k}^{t}-\pi \right)\;\\ \bar{s}\cos\left(\theta_{k}^{t}-\pi \right) \end{array}\right],$$

(57) $${\tilde{J}}_{k}\left(\theta_{k}^{t}\right)=\left[\begin{array}{cccc} P_{xx} & P_{xy} & 0 & 0\\ P_{yx} & P_{yy} & 0 & 0\\ 0 & 0 & P_{\dot{x}\dot{x}} & P_{\dot{x}\dot{y}}\\ 0 & 0 & P_{\dot{y}\dot{x}} & P_{\dot{y}\dot{y}} \end{array}\right],$$

where:

(58) $$P_{xx}={\left(\Delta r\right)}^{2}\;\sin^{2}\;\left(\theta_{k}^{t}\;\right)+{\bar{r}}^{2}\sigma_{\theta }^{2}\cos^{2}\;\left(\theta_{k}^{t}\right),$$

(59) $$P_{yy}={\left(\Delta r\right)}^{2}\;\cos^{2}\;\left(\theta_{k}^{t}\;\right)+{\bar{r}}^{2}\sigma_{\theta }^{2}\sin^{2}\;\left(\theta_{k}^{t}\right),$$

(60) $$P_{xy}=P_{yx}=\frac{1}{2}\sin2\theta_{k}^{t}\left[{\left(\Delta r\right)}^{2}-{\bar{r}}^{2}\sigma_{\theta }^{2}\right],$$

(61) $$P_{\dot{x}\dot{x}}=\sigma_{s}^{2}\sin^{2}\left(\theta_{k}^{t}-\pi \right)+\sigma_{c}^{2}{\bar{s}}^{2}\cos^{2}\left(\theta_{k}^{t}-\pi \right),$$

(62) $$P_{\dot{x}\dot{x}}=\sigma_{s}^{2}\cos^{2}\left(\theta_{k}^{t}-\pi \right)+\sigma_{c}^{2}{\bar{s}}^{2}\sin^{2}\left(\theta_{k}^{t}-\pi \right),$$

(63) $$P_{\dot{x}\dot{y}}=P_{\dot{y}\dot{x}}=\frac{1}{2}\sin\left(2\left(\theta_{k}^{t}-\pi \right)\right)\left[\sigma_{s}^{2}-\sigma_{c}^{2}{\bar{s}}^{2}\right].$$

Hence, the elementary symmetric functions are calculated over the set defined by

(64) $$\Lambda_{k}\left(v,Z\right)=\left\{\frac{\langle 1,\kappa_{k}\rangle }{\kappa_{k}\left(z_{k}\right)}\left(\frac{\omega_{k}^{b}}{V_{H}}+C_{k}P_{D,k}\sum_{i=1}^{J_{k-1}}\sum_{a=1}^{A_{k}}\omega_{k|k-1}^{\left(i\right)}\lambda_{k,a}q_{k}^{\left(i\right)}\left({\hat{z}}_{k,a}\right)\right):z\in Z\right\}.$$

The inner products involved in the CPHD recursions are obtained by

(65) $$\begin{array}{c} \langle 1,v_{k|k-1}\rangle =\int \sum_{i=1}^{J_{k-1}}\omega_{k|k-1}^{\left(i\right)}N\left(x;{\hat{x}}_{k|k-1}^{\left(i\right)},J_{k|k-1}^{\left(i\right)}\right)+\Psi \left(x;\gamma_{k}\left(\theta ,r,c,s\right)\right)dx\;\\ \;\;=\langle 1,\omega_{k|k-1}\rangle +\omega_{k}^{b}, \end{array}$$

(66) $$\langle 1-P_{D,k},v_{k|k-1}\rangle =\sum_{i=1}^{J_{k-1}}\left[1-P_{D,k}\right]\omega_{k|k-1}^{\left(i\right)}=\left[1-P_{D,k}\right]\langle 1,\omega_{k|k-1}\rangle ,$$

(67) $$Y_{k}^{u}\left[v,Z\right]\left(n\right)=\sum_{i=0}^{\min\left(\left|Z\right|,n\right)}\;\;\left(\left|Z\right|-i\right)!p_{\kappa ,k}\left(\left|Z\right|-i\right)P_{i+u}^{n}{\left[1-P_{D,k}\right]}^{n-\left(i+u\right)}\frac{{\langle 1,\omega_{k|k-1}\rangle }^{n-\left(i+u\right)}}{{\left(\langle 1,\omega_{k|k-1}\rangle +\omega_{k}^{b}\right)}^{n}}E_{i}\left(\Lambda_{k}\left(v,Z\right)\right).$$

Finally, the posterior PHD is approximated by

(68) $$\begin{array}{c} v_{k|k}\left(x,\beta =0\right)=\left[1-P_{D,k}\right]\chi v_{k|k-1}\left(x,\beta =0\right)+\sum_{z_{k}\in Z^{k}}\chi \left(z\right)\psi_{k}\left(x,\beta =0\right)v_{k|k-1}\left(x,\beta =0\right)\\ \;=\left[1-P_{D,k}\right]\chi \sum_{i=1}^{J_{k-1}}\omega_{k|k-1}^{\left(i\right)}N\left(x;{\hat{x}}_{k|k-1}^{\left(i\right)},J_{k|k-1}^{\left(i\right)}\right)\\ \;+\sum_{z_{k}\in Z^{k}}\chi \left(z\right)\psi_{k}\left(x,\beta =0\right)\sum_{i=1}^{J_{k-1}}\omega_{k|k-1}^{\left(i\right)}N\left(x;{\hat{x}}_{k|k-1}^{\left(i\right)},J_{k|k-1}^{\left(i\right)}\right)\\ \;\approx \left[1-P_{D,k}\right]\chi \sum_{i=1}^{J_{k-1}}\omega_{k|k-1}^{\left(i\right)}N\left(x;{\hat{x}}_{k|k-1}^{\left(i\right)},J_{k|k-1}^{\left(i\right)}\right)\\ \;+\sum_{z_{k}\in Z^{k}}\sum_{i=1}^{J_{k-1}}\frac{\langle 1,\kappa_{k}\rangle }{\kappa_{k}\left(z_{k}\right)}\chi \left(z\right)P_{D,k}\omega_{k|k-1}^{\left(i\right)}C_{k}\sum_{a=1}^{A_{k}}\lambda_{k,a}N\;\left({\hat{z}}_{k,a};Hx_{k},R_{k,a}\right)N\left(x;{\hat{x}}_{k|k-1}^{\left(i\right)},J_{k|k-1}^{\left(i\right)}\right)\\ \;=\left[1-P_{D,k}\right]\chi \sum_{i=1}^{J_{k-1}}\omega_{k|k-1}^{\left(i\right)}N\left(x;{\hat{x}}_{k|k-1}^{\left(i\right)},J_{k|k-1}^{\left(i\right)}\right)\\ \;+\sum_{z_{k}\in Z^{k}}\sum_{i=1}^{J_{k-1}}\sum_{a=1}^{A_{k}}\frac{\langle 1,\kappa_{k}\rangle }{\kappa_{k}\left(z_{k}\right)}\chi \left(z\right)P_{D,k}C_{k}\omega_{k|k-1}^{\left(i\right)}\lambda_{k,a}N\;\left({\hat{z}}_{k,a};Hx_{k},R_{k,a}\right)N\left(x;{\hat{x}}_{k|k-1}^{\left(i\right)},J_{k|k-1}^{\left(i\right)}\right)\;\\ \;=\sum_{i=1}^{J_{k-1}}\omega_{k,m}^{\left(i\right)}N\left(x;{\hat{x}}_{k|k-1}^{\left(i\right)},J_{k|k-1}^{\left(i\right)}\right)\;\\ \;+\sum_{z_{k}\in Z^{k}}\sum_{i=1}^{J_{k-1}}\sum_{a=1}^{A_{k}}\frac{\langle 1,\kappa_{k}\rangle }{\kappa_{k}\left(z_{k}\right)}\chi \left(z\right)P_{D,k}C_{k}\omega_{k|k-1}^{\left(i\right)}\lambda_{k,a}q_{k}^{\left(i\right)}\left({\hat{z}}_{k,a}\right)N\left(x;{\hat{x}}_{k|k}^{\left(i,a\right)},J_{k|k}^{\left(i,a\right)}\right)\;\\ \;=\sum_{i=1}^{J_{k-1}}\omega_{m,k}^{\left(i\right)}N\left(x;{\hat{x}}_{k|k-1}^{\left(i\right)},J_{k|k-1}^{\left(i\right)}\right)+\sum_{z_{k}\in Z^{k}}\sum_{i=1}^{J_{k-1}}\sum_{a=1}^{A_{k}}\omega_{s,k}^{\left(i,a\right)}N\left(x;{\hat{x}}_{k|k}^{\left(i,a\right)},J_{k|k}^{\left(i,a\right)}\right), \end{array}$$

and

(69) $$\begin{array}{c} v_{k|k}\left(x,\beta =1\right)=\sum_{z_{k}\in Z^{k}}\chi \left(z\right)\psi_{k}\left(x,\beta =1\right)v_{k|k-1}\left(x,\beta =1\right)\;\\ \;=\sum_{z_{k}\in Z^{k}}\chi \left(z\right)\frac{\langle 1,\kappa_{k}\rangle }{\kappa_{k}\left(z_{k}\right)}g_{k}\left(z_{k}|x\right)P_{D,k}\left(x,\beta =1\right)\Psi \left(x;\gamma_{k}\left(\theta ,r,c,s\right)\right)\;\\ \;\approx \sum_{z_{k}\in Z^{k}}\frac{\langle 1,\kappa_{k}\rangle }{\kappa_{k}\left(z_{k}\right)}\chi \left(z\right)\frac{\omega_{k}^{b}}{2\pi }\sum_{a=1}^{A_{k}}\lambda_{k,a}N\left(x;{\tilde{x}}_{k}\left(\theta_{k}^{t}\right),{\tilde{J}}_{k}\left(\theta_{k}^{t}\right)\right)\;\\ \;=\sum_{z_{k}\in Z^{k}}\sum_{a=1}^{A_{k}}\frac{\langle 1,\kappa_{k}\rangle }{\kappa_{k}\left(z_{k}\right)}\chi \left(z\right)\frac{\omega_{k}^{b}}{2\pi }\lambda_{k,a}N\left(x;{\tilde{x}}_{k}\left(\theta_{k}^{t}\right),{\tilde{J}}_{k}\left(\theta_{k}^{t}\right)\right)\;\\ \;=\sum_{z_{k}\in Z^{k}}\sum_{a=1}^{A_{k}}\omega_{b,k}^{\left(a\right)}N\left(x;{\tilde{x}}_{k}\left(\theta_{k}^{t}\right),{\tilde{J}}_{k}\left(\theta_{k}^{t}\right)\right), \end{array}$$

where

(70) $$\omega_{m,k}^{\left(i\right)}=\left[1-P_{D,k}\right]\chi \omega_{k|k-1}^{\left(i\right)},$$

(71) $$\omega_{s,k}^{\left(i,a\right)}=\frac{\langle 1,\kappa_{k}\rangle }{\kappa_{k}\left(z_{k}\right)}\chi \left(z\right)P_{D,k}C_{k}\omega_{k|k-1}^{\left(i\right)}\lambda_{k,a}q_{k}^{\left(i\right)}\left({\hat{z}}_{k,a}\right),$$

(72) $$\omega_{b,k}^{\left(a\right)}=\frac{\langle 1,\kappa_{k}\rangle }{\kappa_{k}\left(z_{k}\right)}\chi \left(z\right)\frac{\omega_{k}^{b}}{2\pi }\lambda_{k,a}.$$

In GMMbCPHD, the component management is same as that of GMMbPHD, while the state extraction is quite different as that of the GMMbPHD. Since the cardinality distribution has been estimated in the GMMbCPHD, the estimated target states can be extracted based on this cardinality distribution. The detailed extraction implementation procedure is: if the cardinality with *N* has the biggest probability in the distribution, *N* target states are extracted from the posterior intensity with the *N* biggest weights.

## 4. Simulation Experiments

For the sake of fair comparison as well as not losing generality, the simulation scenarios follow the exact same as those in [17]. The improvements of cardinality and localization estimation of GMMbCPHD are presented by comparing with GMbPHDwEKF, GMbPHDwUKF, GMMbPHD, GMbCPHDwEKF as well as GMbCPHDwUKF.

### 4.1. Simulation Scenarios

#### 4.1.1. Experiment 1

**Figure 1:**
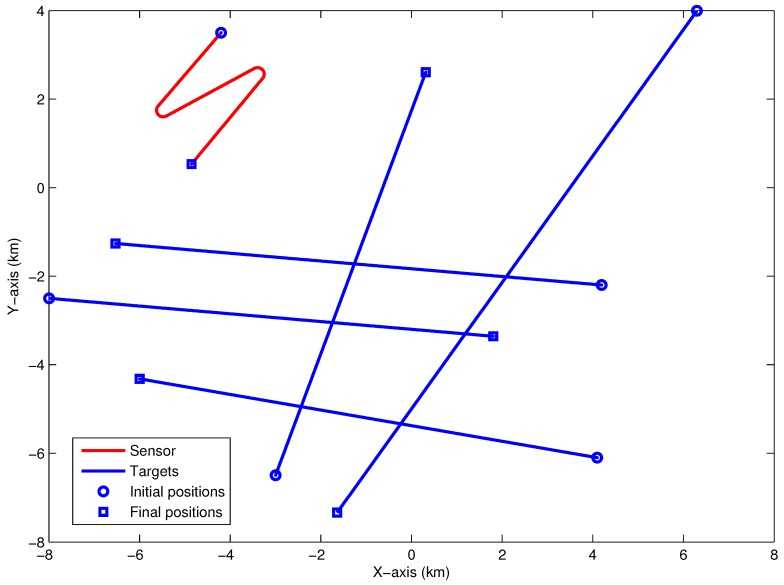
The geometry between sensor and targets (the original source of this picture is from Figure 2 in [17]).

The initial position of the bearings-only sensor installed in a maneuvering moving platform is $\left[-4200\;m,3500\;m\right]$. To satisfy the observability condition, the sensor platform firstly moves at a speed of 5 knots and changes its course twice: the first one changes from $220^{\circ }$ to $60^{\circ }$ from the time 840th seconds to 1360th seconds, and the second time changes from $60^{\circ }$ to $220^{\circ }$ from the time 1860th seconds to 2040th seconds. In this paper, we define the positive direction of measurements is that the clockwise rotation from the positive Y-axis. In different periods, the number of targets changes along with time and Table 1 shows the motions of all targets. Targets #1, #2, #3, #4, #5 start to move from the initial point $\left[-8000,-2500\right]\;m$, $\left[-3000,\;-6500\right]\;m$, $\left[4100,\;-6100\right]\;m$, $\left[4200,\;-2200\right]\;m$ and $\left[6300,\;4000\right]\;m$, respectively. Figure 1 presents the moving condition of the sensor and the targets.

The simulation lasts 3000 s and the sampling interval is 10 s. Suppose that the measurement noise has the standard deviation $\sigma_{\theta }=1^{\circ }$, and the survival probability of each target is $P_{S,k}=0.98$. To simulate the practical situations, we assume that the clutter distributes uniformly in the measurement space and the number follows the Poisson distribution with mean 15. As each target cannot be detected at each sampling time, the detection probability is assumed to be 0.95. For the new born targets, the prior target course and speed are set to be $\theta_{k}^{t}-\pi$ and $\bar{s}=10$ knots with standard deviation $\sigma_{c}=50^{\circ }$ and $\sigma_{s}=4$ knots, respectively. Usually, the initial target range information is barely known, for simplicity, the initial target range information required in the UKF-based and EKF-based algorithm is assumed to follow a Gaussian distribution in this simulation, with known mean $\bar{r}$ = 12,000 m and standard deviation $\sigma_{r}=4000$ m and, the minimum and maximum target range information required in the GMMbPHD and GMMbCPHD are set to be $\left[300\;m,\;18,000\;m\right]$, respectively. The number of measurement components at each scan is predefined to be $A_{k}=8$. In each filter, the birth intensity is assumed to be $\omega_{k}^{b}=0.05$. and the biggest number of Gaussian components is $M_{k}=100$.

#### 4.1.2. Experiment 2

**Figure 2:**
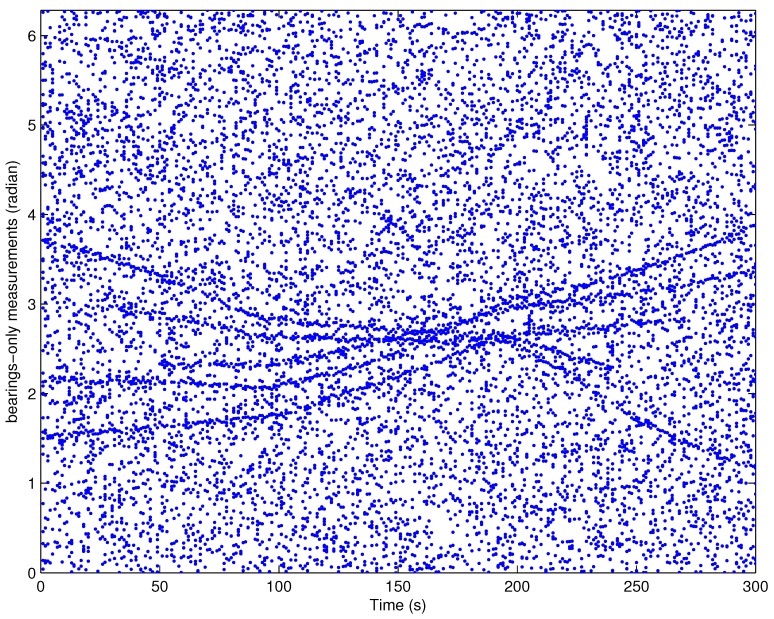
A demonstration of sensor received measurements (the original source of this picture is from Figure 3 in [17]).

To show the advantages of proposed filter in more challenging situation, this experiment considers a higher clutter measurement density as well as a lower target detection probability compared to the experiment 1, i.e., the averaged number of clutter measurements increases to 30 while the target detection probability decreases to 0.85. All remained parameters are exactly same as those in experiment 1. Figure 2 presents the demonstration of the measurements for experiment 2.

#### 4.1.3. Experiment 3

Some preselected parameters for each filter are changed in this simulation experiment. The survival probability $P_{S,k}$ and the birth intensity $\omega_{k}^{b}$ are changed to 0.95 and 0.01, respectively. Furthermore, the biggest number of Gaussian components $M_{k}$ increases to 150. The remained parameters in this simulation experiment are same as those in experiment 1.

#### 4.1.4. Experiment 4

**Figure 3:**
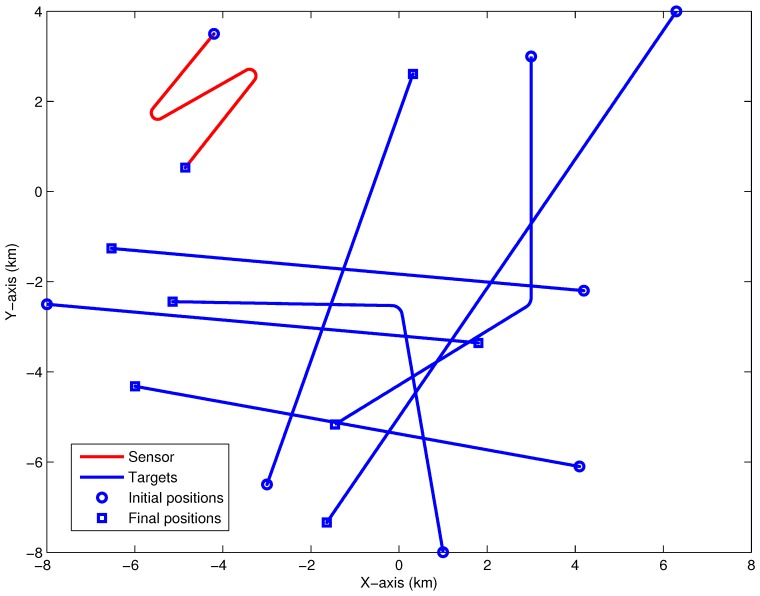
The geometry between sensor and targets (the original source of this picture is from Figure 4 in [17])).

To illuminate the effectiveness and superiority of our proposed method in the scenario of maneuvering target tracking, this experiment extra adds another two maneuvering targets (Targets #6and#7) to the scenario in experiment 1, as depicted in Figure 3. Targets #6and#7 are birth in the point $\left[1000\;m,-8000\;m\right]$ and $\left[3000\;m,3000\;m\right]$, respectively. The traveling courses of Target #6 is changed from $350^{\circ }$ to $270^{\circ }$ at 1500th seconds and from $180^{\circ }$ to $240^{\circ }$ at 1680th seconds for target #7. Targets #6 survives from 200th seconds to 2800th seconds, and target #7 survives from 400th seconds to 3000th seconds. The speeds of both maneuvering targets are 8 knots.

### 4.2. Simulation Results

**Figure 4:**
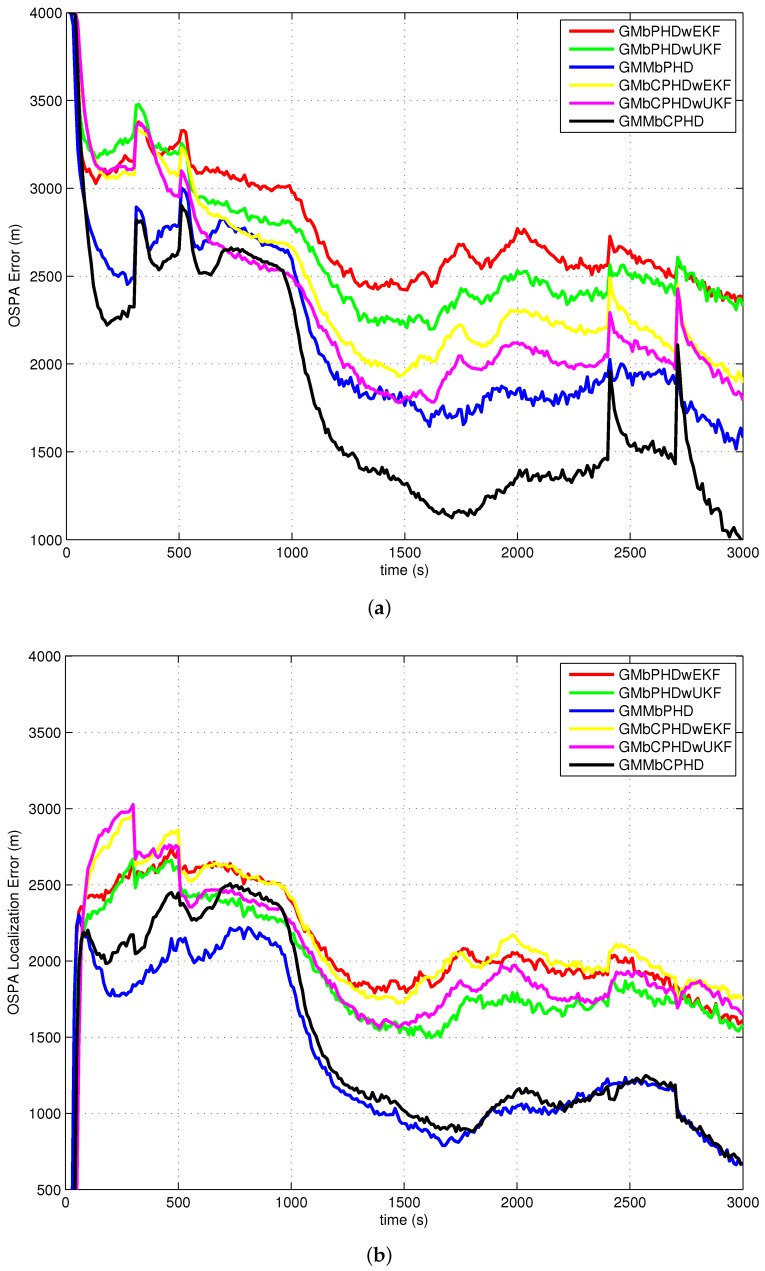

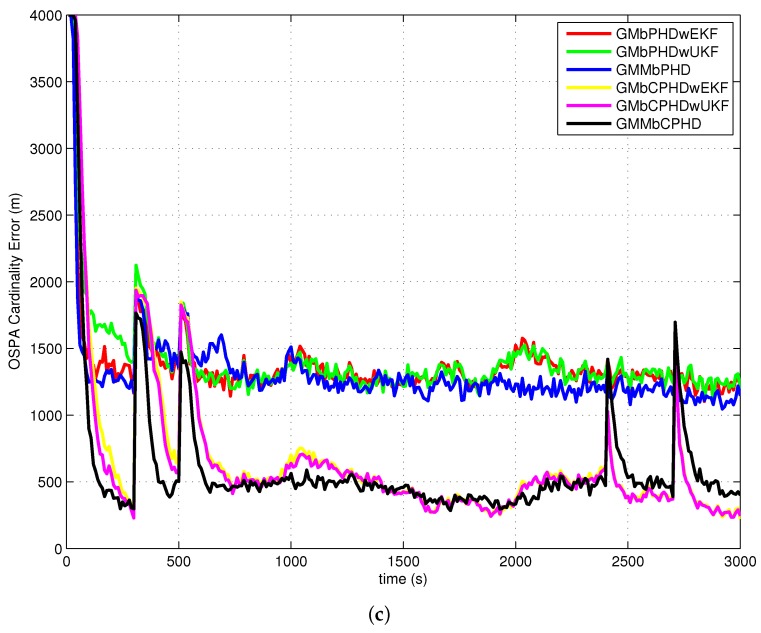
Results of experiment 1: (**a**) OSPA error; (**b**) OSPA localization error; (**c**) OSPA cardinality error.

**Figure 5:**
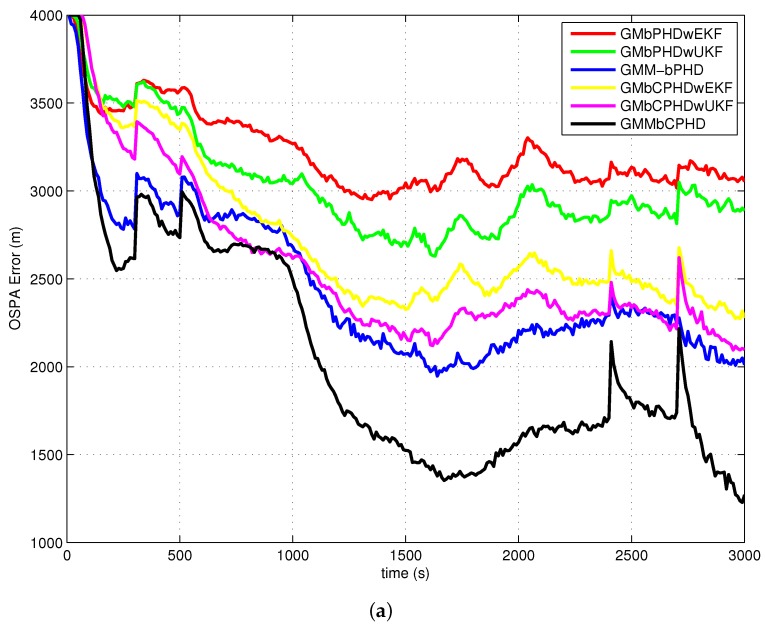

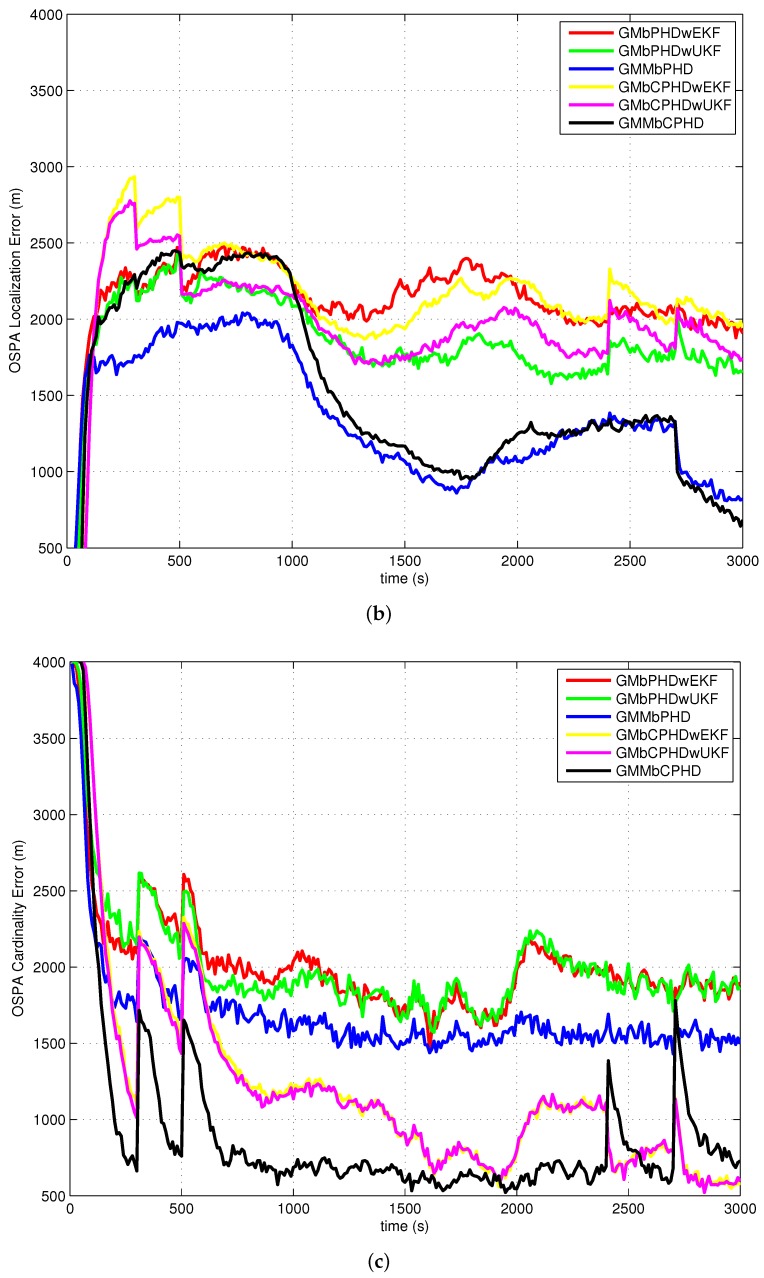
Results of experiment 2: (**a**) OSPA error; (**b**) OSPA localization error; (**c**) OSPA cardinality error.

**Figure 6:**
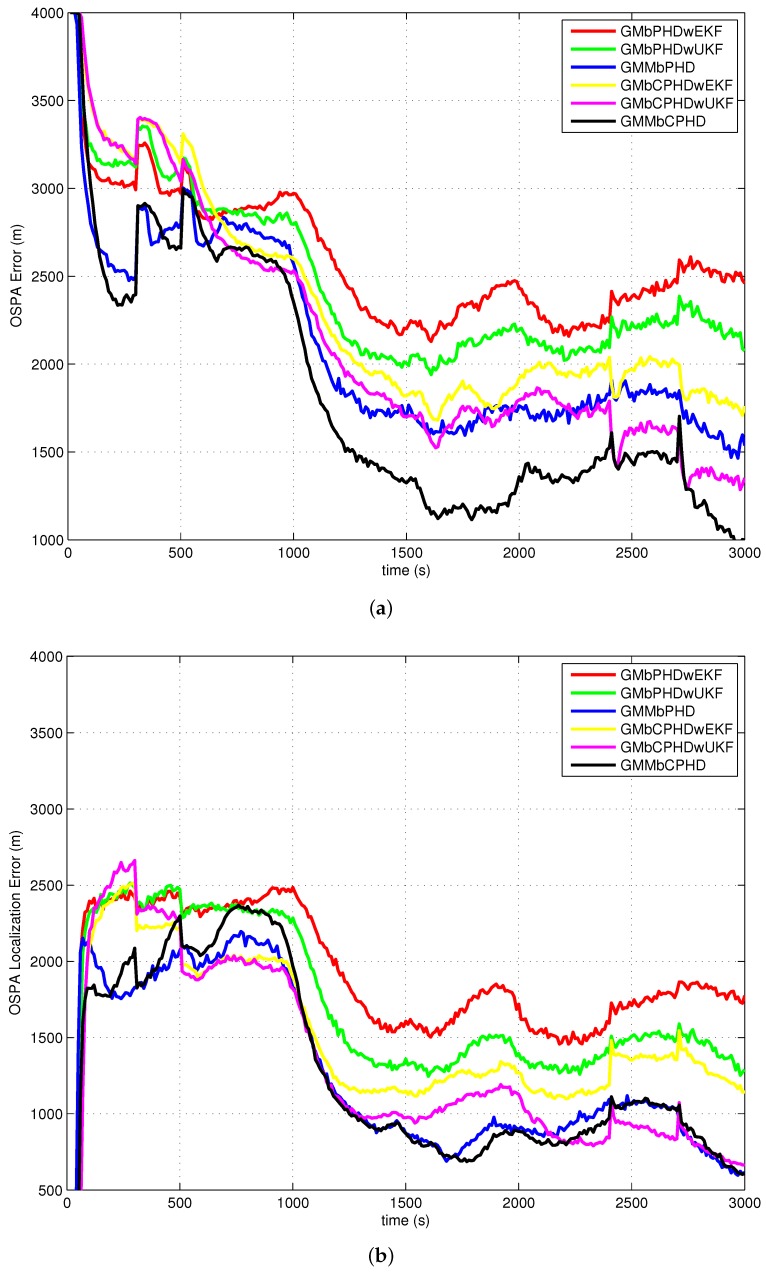

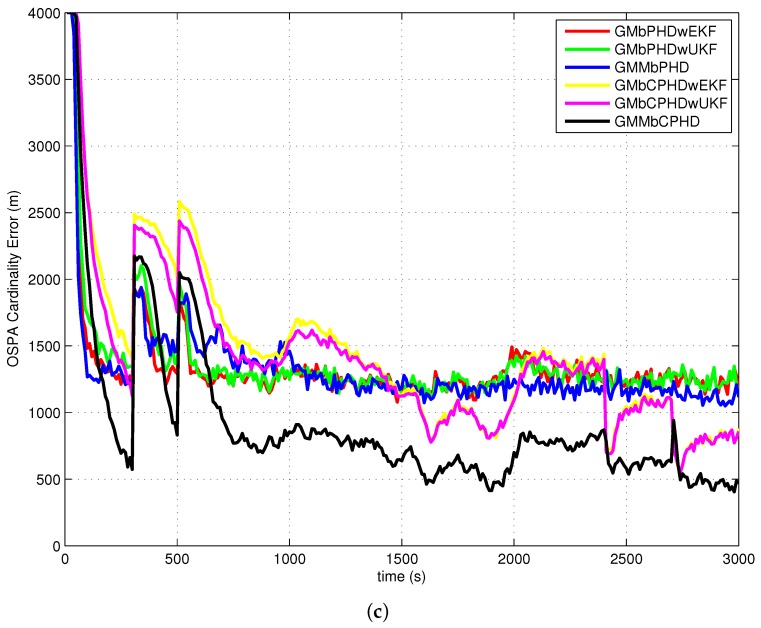
Results of experiment 3: (**a**) OSPA error; (**b**) OSPA localization error; (**c**) OSPA cardinality error.

**Figure 7:**
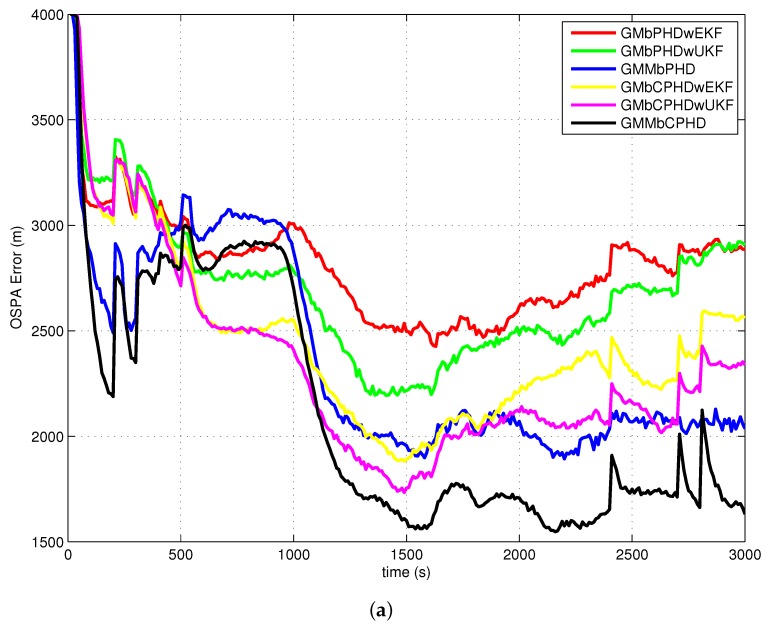

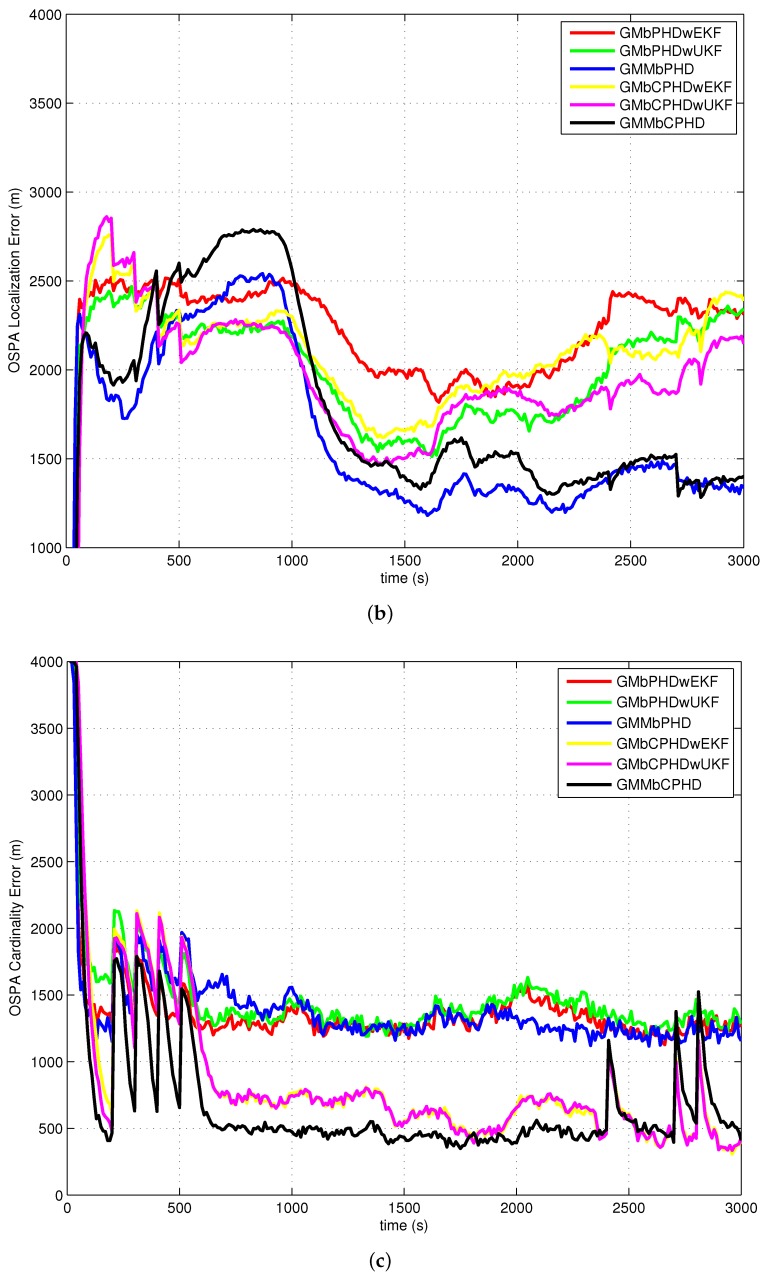
Results of experiment 4: (**a**) OSPA error; (**b**) OSPA localization error; (**c**) OSPA cardinality error.

**Figure 8:**
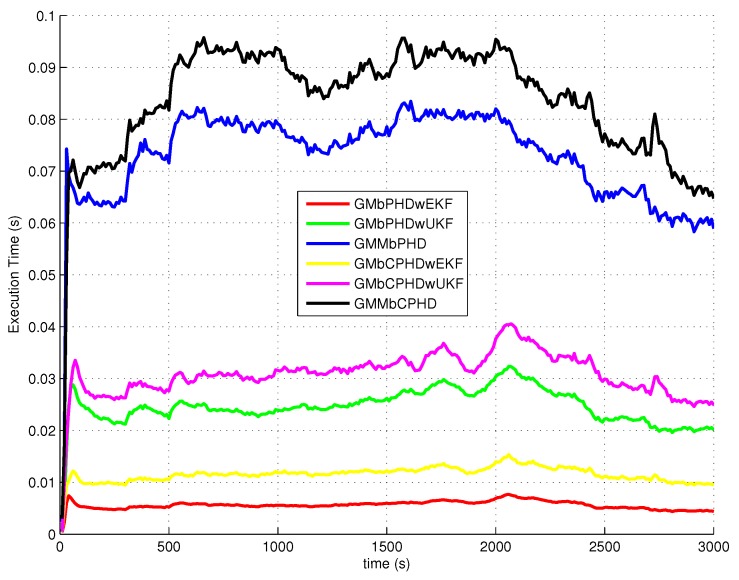
Execution time.

The average OSPA with order 2 and cutoff 400 is used as a metric to evaluate performances of each filter over 500 Monte Carlo runs. The simulation results of experiment 1, experiment 2, experiment 3 and experiment 4 are shown in Figure 4, Figure 5, Figure 6 and Figure 7, respectively. Basically, the OSPA error consists of two parts (OSPA localization error and OSPA cardinality error). To fully show the benefits of the filters, both the OSPA distance and the OSPA components (OSPA localization and cardinality) are also shown in each experiment result. The execution time to show the computational load of each filter in experiment 1 is given in Figure 8.

In all simulation experiments, as can be seen obviously from Figure 4a, Figure 5a, Figure 6a and Figure 7a, the proposed GMMbCPHD filter delivers much lower OSPA distance error than other filters. As the nonlinear bearings-only measurement is approximated by Gaussian mixtures using GMM technique which is more accurate than that is approximated by a single Gaussian distribution, the localization estimation of targets in GMMbPHD and GMMbCPHD is much more accurate than EKF and UKF-based filters, such as GMbPHDwEKF, GMbPHDwUKF, GMbCPHDwEKF and GMbCPHDwUKF as shown in Figure 4b, Figure 5b, Figure 6b and Figure 7b. From Figure 4c, Figure 5c, Figure 6c and Figure 7c, we can see that the CPHD-based filters deliver much lower OSPA cardinality error than those of PHD-based filters such as GMbPHDwEKF, GMbPHDwUKF and GMMbPHD as the cardinality distribution has been modeled and propagated properly in GMbCPHDwEKF, GMbCPHDwUKF and GMMbCPHD.

Compared results of experiment 1 and experiment 2, we can see that the OSPA distance, OSPA localization and OSPA cardinality in experiment 1 are more accurate than those in experiment 2. As experiment 2 has much higher clutter density and lower target detection probability than those in experiment 1, the tracking situation in experiment 2 is more challenging than that in experiment 1. Fortunately, the proposed GMMbCPHD also performs much better than other filters. From results of experiment 3, we can see that GMMbCPHD has stable performance than other filters as the changed preselected parameters do not have much influence on the the proposed filter. However, these preselected parameters have negative effects on filters such as GMbCPHDwEKF and GMbCPHDwUKF. When two extra maneuvering targets are considered, the OSPA errors of all filters are increased as shown in Figure 7. However, the proposed GMMbCPHD also shows the best performance compared with other filters.

In all simulation experiments, all compared filters present a large OSPA distance error and OSPA localization error before 1000 s as the target states are not observable. After the maneuvering motion of sensor, the observability condition is satisfied and the OSPA distance and localization errors decrease sharply. Based on the discussion above, the improvements of proposed GMMbCPHD reflect in both target localization estimation and target cardinality estimation. As GMM technique can improve the localization tracking accuracy, the GMMbPHD and GMMbCPHD show advantages on the OSPA localization. Besides, estimating cardinality distribution can improve the cardinality estimation of multitarget states and the GMMbCPHD gives the benefits on OSPA cardinality. However, the GMM technique and cardinality distribution estimation will increase the computational load of the filter. Figure 8 shows the execution times of all filters and we can see that GMMbPHD and GMMbCPHD filters have a little bit heavier load than other filters. As the measurement likelihood function is modeled by single Gaussian distribution in GMbCPHDwEKF and the likelihood function of each measurement is modeled using eight Gaussian measurement components in proposed GMMbCPHD, the computational time of GMMbCPHD is almost eight times heavier than that of GMbCPHDwEKF as shown in Figure 8. Please note that each measurement component is used to update predicted intensity component using Kalman filter. Similarly, the GMbCPHDwUKF approximates the measurement likelihood function using nine sigma points, but these sigma points are utilized in only one Kalman filter cycle. Thus, the computational time of GMbCPHDwUKF just shows a little bit heavier than that of GMbCPHDwEKF. For simulation scenarios with more number of targets or clutter measurements, the computational loads of all methods obviously increase as more intensity components will be generated and more Kalman filter cycles are performed in algorithms. Though the GMM technique takes much computational sources in the GMM-based filters, fortunately, the execution time of proposed GMMbCPHD shows much smaller than the real time. Please note that all simulation cases are executed on the platform with a 2.4 GHz Intel Core i5 CPU, Windows 7, and Matlab.

## 5. Conclusions

In this paper, we proposed a random finite set (RFS)-based filter, Gaussian mixture measurements-based cardinality probability hypothesis density (GMMbCPHD), which delivers significant benefits from two aspects: Gaussian mixture measurements (GMM) and cardinality distribution estimation. The GMM models the nonlinear bearings-only measurement likelihood using Gaussian mixtures but not single Gaussian distribution. The GMM is used in the proposed GMMbCPHD and each measurement component is used to update the predicted intensity components. Thus, the number of updated intensity components is also larger than that in GMbCPHDwEKF and GMbCPHDwUKF. In traditional GMbCPHD, the likelihood function is modeled by single Gaussian distribution; however, that is approximated using Gaussian mixtures in GMMbCPHD. Each measurement is associated with each predicted intensity component with state updated using Kalyan filter. Furthermore, the predicted cardinality distribution is also updated using measurement components of all received measurement. As the GMM is more precise to model the likelihood function, the estimation of updated intensity and cardinality show significant improvement. The GMMbCPHD also propagates the cardinality distribution estimation which can improve the target number estimation in multitarget tracking. In different simulation scenarios with different parameters, simulation results show that the proposed filter performs much better than other filters on both optimal subpattern assignment (OSPA) localization and OSPA cardinality.

## Figures and Tables

**Table 1:** Motion details of targets.

Target Index	Survival Time (s)	Course (Degree)	Speed (Knots)
#1	$\left[0,\;2400\right]$	95	8
#2	$\left[300,\;3000\right]$	20	7
#3	$\left[500,\;3000\right]$	280	8
#4	$\left[0,\;3000\right]$	275	7
#5	$\left[0,\;2700\right]$	215	10
